# Digital health in South Africa: innovating to improve health

**DOI:** 10.1136/bmjgh-2018-000722

**Published:** 2018-04-24

**Authors:** Yogan Pillay, Pakishe Aaron Motsoaledi

**Affiliations:** HIV, TB and MNCH, National Department of Health, South Africa, Pretoria, Gauteng, South Africa

**Keywords:** maternal health, child health, health education and promotion

In the postapartheid era, South Africa’s public health system has been transformed from a dysfunctional system which perpetuated discrimination based by race and inequality into a deracialised, more comprehensive and integrated health system.^1^ In 2015, rates of utilisation of health services across the continuum of care were the highest in sub-Saharan Africa: 94% of pregnant women received antenatal care (ANC), 76% received the recommended four ANC visits, 96% delivered in a health facility, 97% had births attended by a skilled provider and 84% attended postnatal care within 2 days following birth.^2^ High rates of service utilisation have been underpinned by programmes like MomConnect which aim to empower pregnant and postpartum women with knowledge and bolster the utilisation of health services.

MomConnect was established by the National Department of Health in 2014 to register pregnancies and provide pregnant and postpartum women with twice-weekly health information text messages as well as access to a help desk for queries and feedback.^3 4^ Since its inception, MomConnect has grown to become one of the largest mHealth initiatives globally; by December 2017, it had cumulatively registered over 1.7 million pregnant women in over 95% (3300) of public health facilities nationally to receive short messaging service health information messages. It had also received more than 14 000 spontaneously reported compliments to the help desk—eight times as many as the 1450 complaints received (personal communication Ms Jane Sebidi, Helpdesk Coordinator). The help desk has also impacted positively on improving the quality of care in the health system.^5^ Both complaints and compliments are sent to local coordinators to feedback to the health professional or facility to which they relate. This feedback is important to ensure that complaints are attended to and the complainant provided with report on action taken and that good work is acknowledged and encouraged.

In this journal supplement, we draw from experiences establishing, implementing and evaluating MomConnect in South Africa to provide critical learnings on the use of mobile and digital technology for global health in low-income and middle-income countries. We start with two editorials. The first, led by Dr Garrett Mehl and colleagues reflects on MomConnect from a global perspective, considering its implications for Universal Health Coverage in South Africa and globally.^6^ This perspective highlights two important features of MomConnect which offer a vehicle for pursuing universal health coverage: (1) its pregnancy registration feature and (2) the forethought in developing an interoperable digital health architecture with ‘common good’ elements for an interoperable digital national Health Information System.^6^ Both of these features set MomConnect apart from other digital health programmes, including maternal mobile messaging initiatives implemented elsewhere, and provide an important foundation for informing the future directionality of the programme and accommodating expansion into new areas. The second editorial, written by Dr Joanne Peter, provides a donor perspective on the history and evolution of MomConnect, considering the importance of public–private partnerships, including government stewardship and sustained donor funding.^7^

Following these editorials, we present a series of seven papers. The first two of these papers provide a background on the history of MomConnect and its architecture. In ‘MomConnect: History, evolution, successes and challenges’, Dr Peter Barron and colleagues provide an overview of MomConnect, detailing the groundwork, partnerships, stewardship and financing undertaken to establish and support it.^4^ Additional detail on the programme design, including registration and linkages to the health system, messaging, help desk and monitoring and evaluation features, help to describe key elements of the programme. In the next paper, Dr Chris Seebregts and colleagues provide an overview of the technical platform for MomConnect.^8^ Reliant on the use of open content, MomConnect’s technical platform uses a health information exchange that can connect to any standards-compliant electronic front-end application as well as to any standards-compliant electronic back-end database.^8^ The end result is a platform which was designed and developed from the outset to be scalable and extendable across the country and serves as a reference implementation for South Africa’s national eHealth architecture.^8^

In the next four papers, we shift focus to analysis articles which draw on quantitative and qualitative methods. In the third paper, Dr Amnesty LeFevre and colleagues explore whether the messaging delivery component of MomConnect, including the underlying technical platform, performed as intended.^9^ Through a series of descriptive analyses, they identify dropouts from point of contact with the health system during ANC clinics to successful registration and measure exposure to health information messages.^9^ While over 60% of all pregnant women attending antenatal clinics in the public sector were registered in 2017, there have been limitations in the current registration processes. There have been a large number of dropouts from registration and if rectified this would translate to an increase in coverage by 12%–19%—representing near universal coverage of all pregnant women attending ANC with access to a mobile phone.^9^ Findings from analyses on message delivery suggest that an estimated 80% of intended messages were successfully sent—a figure well above that reported elsewhere for maternal messaging programmes.^10^ In the fourth paper, Dr Alexa Heekes and colleagues demonstrate the feasibility and value of linking MomConnect data with records collected through a province-wide health information exchange in the Western Cape Province of South Africa.^11^ Pregnant women registered with MomConnect had fewer adverse pregnancy outcome events such as stillbirths. However, data suggest that those at lower risk of adverse pregnancy outcomes were more likely to register for MomConnect.^11^

The fifth and sixth papers in the series focus on MomConnect’s helpdesk. In the fifth paper, Ms  Khou Xiong and colleagues explored trends in the utilisation of the helpdesk, determining that 8% of MomConnect’s registered users utilised the helpdesk.^12^ Helpdesk use was primarily to seek maternal health information and where feedback about health services was provided, there were significantly more compliments than complaints.^12^ In the second help desk paper, Dr Matt Engelhard and colleagues evaluate the need for and feasibility of automated message triage to improve help desk responsiveness to high-priority messages.^13^ Using keyword matching and a Naive Bayes classifier, low prevalence high-priority messages related to the disrespect and abuse of women during childbirth were effectively identified, which could support automated triage to improve handling of high-priority messages moving forward.^13^ The seventh and final paper in this series led by Dr Donald Skinner and colleagues explores user perceptions of and the use of MomConnect health information messages through qualitative in-depth interviews and focus group discussions.^14^ Findings suggest that MomConnect users were enthusiastic about the messages, reporting that the information was useful and empowering, with some saving the messages to use as a resource or to share with others.^14^

Finally, we conclude our series with a final editorial on ‘Taking digital health innovation to scale in South Africa: ten lessons from MomConnect’.^15^ In this last paper, Dr Joanne Peter and colleagues highlight lessons across domains of leadership and partnerships, technology and architecture, content and user engagement, financial health and monitoring and evaluation.^15 16^ In view of the limited number of digital health programmes which have been scaled globally and which are government led, the emerging lessons are anticipated to provide vital insights for other countries similarly considering the pros and cons of technology use in the health sector.

This is the first journal supplement of its kind on a national level digital health programme. MomConnect is one of the only five mobile health information messaging programmes to have scaled to over 1 million beneficiaries and the only programme to have attained population level coverage of >60%.^15^ While a multitude of factors underpin the programme’s successful implementation, perhaps none are more important than the pregnant and postpartum women in South Africa who form the backbone of our communities, and the health workers who work tirelessly to register women and provide vital health services. The commitment to technology use in the health sector is a critical component of the Department of Health’s vision to embrace innovative strategies for improving health services. By reflecting on components of MomConnect, we hope to catalyse discussion on how to optimally design, prioritise, plan and successfully implement a new wave of digital health solutions in South Africa and in other low-resource settings where the disease burden is acute.
